# Clinical case of co-infection: Dengue fever and respiratory mycoplasmosis in a child

**DOI:** 10.1016/j.rmcr.2024.102158

**Published:** 2024-12-26

**Authors:** D.V. Preobrazhenskaia, E.V. Melekhina, Zh.B. Ponezheva

**Affiliations:** Central RI [Research Institute] of Epidemiology, Federal Service for the Oversight of Consumer Protection and Welfare (Rospotrebnadzor), Moscow, Russia

**Keywords:** Dengue fever, *M. pneumoniae* infection, Children, Co-infection, Therapy for arboviral infections

## Abstract

According to WHO, dengue fever (DF) is currently endemic to more than 100 countries in various regions of Africa, America, and Asia; outbreaks have been reported in Europe. In the Russian Federation, there is a much smaller proportion of children among those infected due to the imported nature of the infection.

We described a clinical case of imported dengue fever in an adolescent girl in Moscow after a 5-day vacation. Despite the fact that during the examination at the hospital in the Maldives, DENV arbovirus antigen was isolated in the blood by immunochromatographic rapid test, the course of the disease had a number of symptoms that did not conform to the classical course of the disease: catarrhal symptoms, cough, elevated C-reactive protein, and radiographic evidences of right-sided maxillary sinusitis. No improvement in the condition was observed despite the therapy administered. Additional examination confirmed an active infection caused by *M. pneumoniae*. After correction of etiotropic and pathogenetic therapy, the patient was discharged with recovery on day 10 of the disease.

After 4 years since the start of the pandemic, an increase in infectious morbidity, particularly DF, has been observed. The proportion of co-infections is increasing. Co-infection of DF and respiratory mycoplasmosis in children may occur masked as an acute respiratory viral infection (ARVI): with intensification of catarrhal and intoxication syndromes and atypical changes in laboratory parameters. All that complicates clinical and laboratory diagnosis and leads to incorrect administration of starting etiotropic therapy.

## 1 Introduction

Dengue fever (DF) is a viral zooanthroponotic infectious disease with a vector-borne transmission route. It is accompanied by high fever and intoxication, myalgia and arthralgia, and exanthema syndrome. Abnormal laboratory parameters (leukopenia, thrombocytopenia) are characteristic. Sometimes DF may be accompanied by hemorrhagic syndrome.

According to the World Health Organization (WHO), the infection is currently endemic in more than 100 countries in different regions: Africa, the Americas, the Eastern Mediterranean, Southeast Asia and the Western Pacific. About 70 % of the global burden of infection occurs in Asian countries. DF is spreading to new areas, including Europe, causing outbreaks. In 2010, local transmission of the infection was reported for the first time in France and Croatia.

In 2019, a record number of DF cases were reported in the world. All regions were affected; DF transmission was first identified in Afghanistan. In the Americas alone, 3.1 million cases were reported, of which more than 25,000 were severe. A significant number of dengue cases were reported in Asian countries: Bangladesh (101,000), Malaysia (131,000), the Philippines (420,000) and Vietnam (320,000). As of 2021, DF continues to be reported in Brazil, Colombia, Cook Island, Fiji, India, Kenya, Paraguay, Peru, the Philippines, Reunion and Vietnam [1].

According to WHO, DF is the second most common vector-borne infection in the world after malaria. More than 2.5 billion people currently live in the area where DF is spread [2]. The incidence of DF in the world today continues to increase, particularly, the growth is noted in the Caribbean. In this region, the severe course of DF primarily affects children. According to a recent study in Grenada, parental awareness of the causes and symptoms of DF is low despite the implementation of preventive measures [3].

Co-infections are currently relevant. A decrease in infectious morbidity was observed due to the implementation of restrictive measures during the COVID-19 pandemic. However, after 4 years since the start of the pandemic, the worldwide increase in the incidence of mycoplasma infection has been observed. Infection caused by *M. pneumoniae* occurs in various clinical forms: both with respiratory symptoms and extrapulmonary manifestations [4].

## 2 Clinical observation

A 14-year-old patient was admitted on November 21, 2023 to the pediatric department of a multidisciplinary hospital in the central federal district of the Russian Federation on day 4 of the disease with complaints of fever for 4 days up to 40 °C, muscle aches, sore throat, runny nose, dry cough, rash on the extremities, nosebleeds, abdominal pain and liquid stools.

From the history of the disease, it is known that the patient fell ill on November 17, while on vacation in the Maldives. Since November 17, fever up to 40 °C and myalgia were noted. From 2 days onwards, a rare dry cough, sore throat and rash on the extremities joined, prompting the parents to seek medical attention at the local hospital. After examination, a diagnosis of dengue fever was made (confirmed by a positive immunochromatographic rapid test for antigen in the blood). Infusion therapy with saline unbalanced solution such as 0.9 % isotonic sodium chloride solution was performed, as well as symptomatic antipyretic therapy. Upon returning to Moscow on day 3 of the disease, severe abdominal pain and liquid stools up to 3 times, and nosebleed occurred at the airport. In this regard, the patient was examined by a physician and hospitalization was recommended, which the parents refused. The next day, there was a repeated rise in temperature to 38.5 °C, liquid stool once, rash on the distal parts of the extremities with rapid spread to the proximal parts, and catarrhal events persisted. An ambulance crew was called, and the patient was hospitalized in the pediatric infectious disease department.

Epidemiological history. No one in the family is sick. The patient was on vacation in the Maldives from November 11 to November 19, where she developed symptoms of the disease on the 6th day of her stay. Contacts with infectious patients, animals, rodents, birds and raw materials of animal origin are denied. Mosquito bites were reported. Swimming in bodies of water: ocean, swimming pool. Consumption of water from open sources and unboiled water is denied. Meals were eaten at the catering facilities, the hotel restaurant. No antibacterial drugs have been taken in the last 6 months.

Life history. In the first year, the patient grew and developed according to her age. She was vaccinated according to the National Calendar of Prophylactic Immunization, not vaccinated against DF. Previous diseases: ARVI not more than 6 times a year. No known hereditary diseases. The patient is observed by an otolaryngologist with a diagnosis of vasomotor rhinitis. No history of trauma or surgery. No allergic history.

**Fig. 1:**
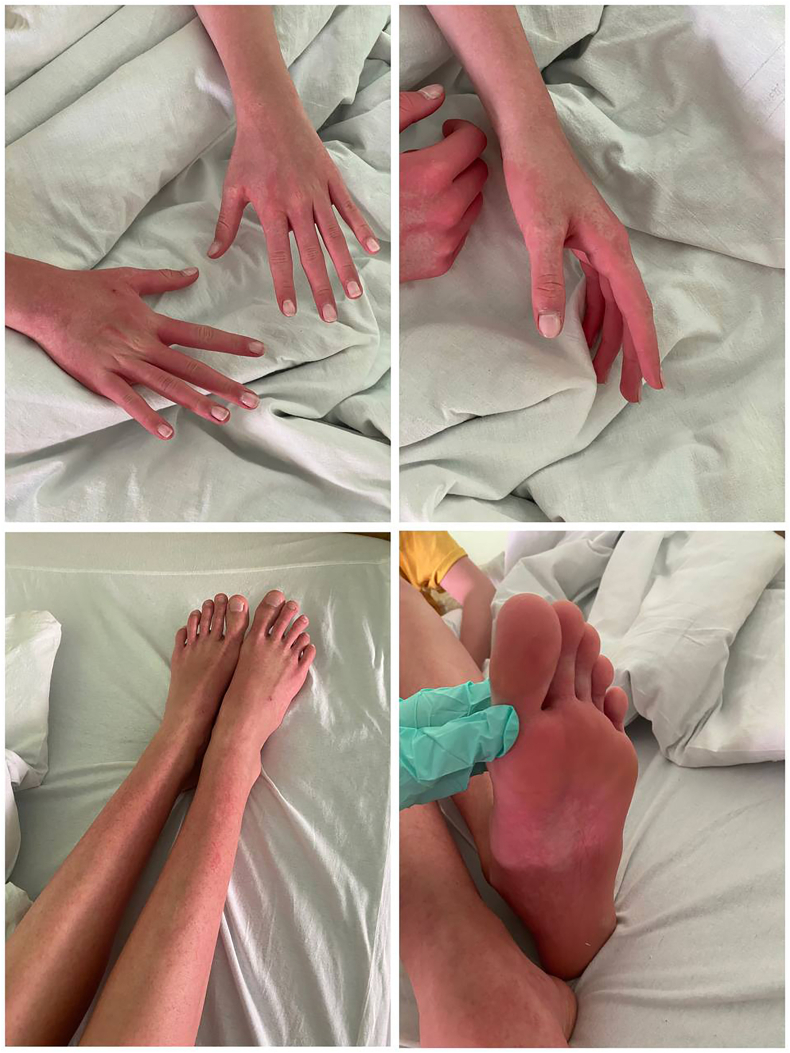
Exanthema syndrome in a patient with dengue fever on day 9 of the disease.

On admission, the condition is moderate due to intoxication and hemorrhagic syndrome. The skin is of normal color, moderately moist. There are elements of a maculopapular rash of the “isles of white in a sea of red” type on the upper and lower extremities and back. The rash is also seen on the feet (see Fig. 1). There are insect bite marks on the upper and lower extremities. The tongue is covered with white plaque at the root. The visible mucous membranes are clean, the posterior pharyngeal wall is moderately hyperemic, there is conjunctival hyperemia in both eyes. Palatine tonsils are Grade 1 enlarged, loose, clean. Cervical lymph nodes are enlarged up to 15 mm, up to 2 per group, painless with soft consistency. Breathing through the nose is moderately difficult, the discharge is mucous. Vesicular breath sounds are heard over the lungs, there are no rales. Heart tones are audible, rhythmic, there are no murmurs. The abdomen is soft, painful on palpation down the intestine, in the epigastric region. Vesical symptoms: Murphy, Kerr, Ortner are negative. The gallbladder point is painless on palpation. The sigmoid colon is not spasmodic. The liver and spleen are not palpable. The genitals are of proper gynecoid form. The stool is liquid, brown, without impurities.

**Fig. 2:**
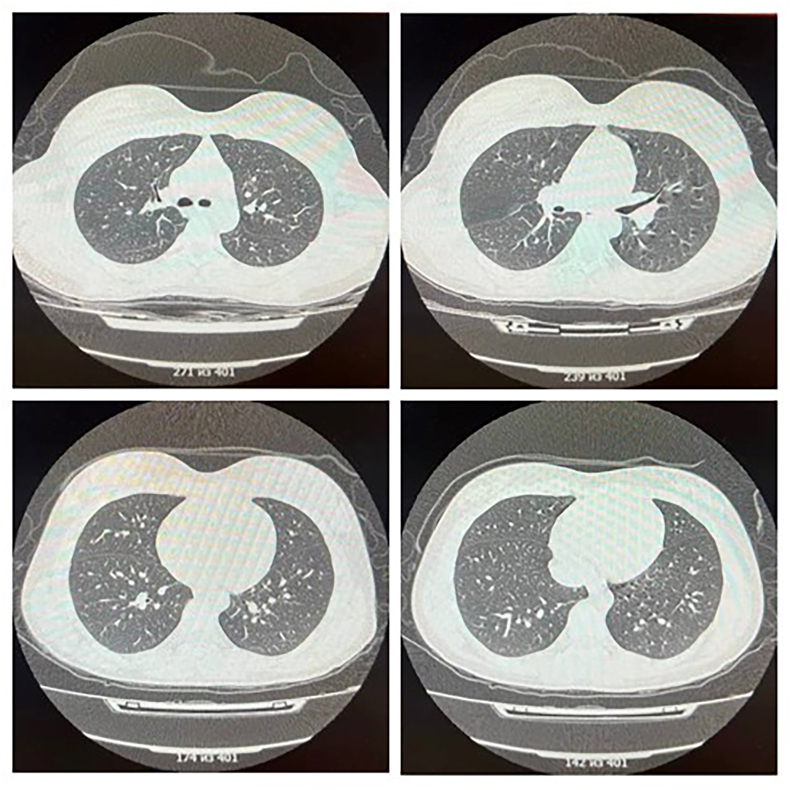
Computed tomography of the chest.

In addition to routine examination, a PCR blood test for dengue virus DNA was performed. For differential diagnostics, examination for malaria and cholera, acute intestinal infections, herpes virus infections, and examination of oropharyngeal swab materials for nucleic acids of 16 respiratory pathogens were performed. The instrumental examinations included abdominal and renal ultrasound scan, thoracic CT scan, ECG, and sinus CT scan (see Fig. 2).

According to the hematology findings (see Table 1), leukopenia, lymphopenia, and thrombocytopenia were noted on admission, which is consistent with changes characteristic of the viral etiology of the disease. In subsequent tests, these changes persisted, and a trend toward greater thrombocytopenia and lymphopenia was noted.

**Table 1:** Hematology panel in the dynamics.

Parameter	November 20 Day 4 of disease	November 21 Day 5 of disease	November 22 Day 6 of disease	November 23 Day 7 of disease	November 24 Day 8 of disease	November 26 Day 10 of disease	Units	Reference limits
Hemoglobin	127	129	135	133	128	122	g/L	115–145
Red blood cell count	4.43	4.48	4.62	4.65	4.54	4.06	10^12^/L	3.80–4.90
Platelet count	**92**↓	**89**↓	**99**↓	**73**↓	**94**↓	272	10^9^/L	150–400
White blood cell count	**2.6**↓	**2.8**↓	**2.5**↓	5.2	**4.4**↓	5.5	10^9^/L	4.5–11.0
Absolute count of neutrophils	**1.4**↓	2.0	1.7	2.9	2.4	3.0	10^9^/L	1.8–7.0
Absolute count of lymphocytes	**0.87**↓	**0.40**↓	**0.63**↓	1.30	1.59	2.05	10^9^/L	0.90–5.00
ESR	–	–	–	–	15	–	mm/h	5–15

According to the blood chemistry findings (see Table 2), C-reactive protein (CRP) level was elevated 2 times of the norm on admission, with subsequent increase to 3 times of the norm in 2 days (by November 22).

**Table 2:** C-reactive protein dynamics.

Parameter	November 20 Day 4 of disease	November 22 Day 5 of disease	November 24 Day 7 of disease	November 26 Day 10 of disease	Units	Reference limits
C-reactive protein	**11.1** ↑	**17.3**↑	4.4	3.8	mg/L	0–5

On the first day of hospitalization on November 20, fever up to 39.6 °C and rash with dynamics to spread to the proximal parts of the extremities persisted. Recurrent nosebleeds were reported. Based on the sinus CT scan findings (signs of bilateral maxillary sinusitis), some dynamics of CRP increase, intensification of catarrhal symptoms, it was decided to start empirical systemic antibacterial therapy (intravenous ceftriaxone). On the second day, November 21, the appearance of skin itching, a single moderate nosebleed, and a rash with gradual spread to the proximal parts of the extremities were noted. The cough syndrome persisted. The body temperature rose to 37.6 °C during the day. Due to the change in the nature of the skin process (itching occurrence), persisting cough, and rhinosinusitis according to CT scan, the material of an oropharyngeal swab was examined to isolate *M. pneumoniae* DNA, and the blood was tested to determine the level of *M. pneumoniae*-specific IgM and IgG.

On the third day of stay, November 22, subfebrile fever and low-productive cough persisted, and skin itching was less troubling on antihistamine therapy. Rash on the hands had a trend towards regression, no nosebleeds were noted in 24 hours.

Taking into account the data of additional examination (positive *M. pneumoniae* IgM), it was decided to change the antibacterial drug (ceftriaxone to oral clarithromycin), intramuscular Meglumine acridonacetate, a low molecular weight interferon inducer, with a wide range of biological activity (antiviral, immunomodulatory, anti-inflammatory) was added at a dose of 250 mg/day every other day, No. 5 [5]. During the nucleic acid amplification test of the whole blood dated November 24, 2023, the RNA of antigenic group B arbovirus, a causative agent of dengue fever, was isolated. The next two days the patient was not feverish, the rash showed a trend towards fading and decreasing itching, and the catarrhal symptoms regressed. The patient was discharged from the Infectious Diseases Department on November 27 feeling good, her condition was satisfactory.

For detoxification and to control intoxication syndrome, the patient received infusion therapy with Meglumine sodium succinate, a balanced polycomponent crystalloid solution, at a dose of 400 mL/day by intravenous drip slowly from the 1st to the 5th day of hospitalization. Meglumine sodium succinate contains Na, K, Mg, Cl ions and succinic acid (succinate), it is an antihypoxic and detoxifying agent widely used in endogenous and exogenous intoxications of various genesis in adults and children from the age of 1 year. Due to the fact that Meglumine sodium succinate solution contains succinate, a universal metabolic intracellular metabolite, the solution has a positive effect on aerobic processes in the cell, reducing the production of free radicals and restoring the energy potential of cells [6], which is extremely relevant in the treatment of infectious diseases.

Thus, based on the data of the disease history, epidemiological anamnesis, complaints, physical and laboratory-instrumental examination data, the final diagnosis was established. The primary diagnosis: dengue fever of moderate severity. The secondary diagnosis: mycoplasma infection (*M. pneumoniae*) of moderate severity. The patient was discharged from the Pediatric Infectious Disease Department on November 27, 2023. On the therapy conducted, symptoms of general intoxication were managed, the temperature normalized, catarrhal events and rash resolved. Harsh breathing persisted in the lungs, there were no rales. Physiological functions were not impaired. Other organs and systems had no negative trends. The patient was discharged home with clinical improvement and recommendations for further treatment.

## 3 Discussion

In the COVID-19 postpandemic period, a change in the usual epidemiology of respiratory tract pathogens has been documented. In addition, the increasing number of tourist trips of Russian Federation residents to countries with tropical and subtropical climates makes the problem of diagnosing infections uncharacteristic of our climate zone extremely important.

In contrast to endemic regions, the imported nature of DF cases determines the age composition of those who became ill with this infection in the Russian Federation, namely a much smaller proportion of children. According to a retrospective study conducted in the Russian Federation, 16 (4.1 %) children aged 1–18 years were among patients with DF during the period of 2009–2019. Of these, hemorrhagic DF was diagnosed in one child aged 9 years [3].

In children, DF often occurs masked as an acute respiratory infection. That is, it is accompanied by fever, hyperemia of the posterior pharyngeal wall, rhinitis and cough. In addition to the catarrhal syndrome in children aged 1 year, the intoxication syndrome is more pronounced, manifested by fever, impaired consciousness, refusal to eat, repeated vomiting. Hepatomegaly is commonly reported [7]. Although most infected infants are asymptomatic. Despite the view that DF in newborns is virtually uncommon due to the presence of maternal antibodies during the first 6 months of life, there are reports of “symptomatic” forms of the disease [8]. Hemorrhagic syndrome in infants is more often manifested by gastrointestinal bleeding. Also, DF can occur in children aged 1 year along with neurological disorders: intracerebral hemorrhages, acute focal or disseminated encephalitis, or encephalomyelitis [9].

In Russia, according to the same retrospective study, the proportion of DF patients for the period of 2009–2019 was 14.9 % of all hospitalized with febrile diseases developed after international travel. Hemorrhagic syndrome in DF was reported in 15.7 % of patients. Prior to the COVID-19 pandemic in Russia, a steady upward trend in the number of verified imported DF cases was observed. This was due to the increase in international tourism and the introduction of DF specific diagnostic methods into clinical practice. Over the ten-year period of observation, the number of cases increased from single cases to a level exceeding the rates of a number of natural focal infections endemic to the Russian Federation [10].

Thus, cases of DF in the Russian Federation are imported and are characteristic of the adult population. The available literature describes several clinical observations of DF in children in the Russian Federation by 2021 at the latest [11]. However, no cases of co-infection have been described. According to researchers from Mexico between 2012 and 2017, there was a decreasing trend in the incidence of severe DF among children aged 0–10 years and an increasing trend in children over the age of 10 years. At the same time, the severity of DF did not change significantly with increasing age [12].

A 14-year-old patient under our follow-up reported clinical manifestations characteristic of DF: febrile intoxication syndrome, myalgia and arthralgia, exanthema syndrome, hemorrhagic syndrome. We also recorded symptoms characteristic of mycoplasma infection: febrile intoxication syndrome, low-productive cough, nasal congestion and mucous discharge from the nose. It was decided to examine the patient for mycoplasma infection due to the negative clinical course of catarrhal symptoms (increased cough, nasal congestion and the amount of mucopurulent nasal discharge), following the results of a consultation with an otorhinolaryngologist. In addition to clinical data, the decision was based on the data of laboratory and instrumental examinations: sinus X-ray (swelling of the mucous membrane of the right maxillary sinus), C-reactive protein increase in dynamics (from 11.1 to 17.3 mg/L). According to the results of examination for mycoplasma infection, positive IgM to *M. pneumoniae* were detected and it was decided to change the antibacterial drug to clarithromycin, an antibiotic from the macrolides group, despite the fact that *M. pneumoniae* DNA was not detected during the PCR examination of the oropharyngeal swab.

Inclusion of Meglumine sodium succinate infusion solution in the pathogenetic treatment program during 5 days of hospitalization contributed to the reduction of systemic inflammatory response (leukocytosis and CRP) and reduced the duration of the syndrome and intoxication in a patient diagnosed with dengue fever of moderate severity due to the complex effect of the solution (antihypoxic, antioxidant and detoxification effects).

## 4 Conclusions

Prior to the COVID-19 pandemic in Russia, a steady upward trend in the number of verified imported DF cases was observed. With the introduction of restrictive measures, there was a decrease in overall infectious morbidity. After 4 years since the start of the pandemic, an increase in infectious morbidity, including DF, has been observed. The proportion of co-infections is also increasing. Co-infection of DF and infections caused by *M. pneumoniae* in children may occur masked as ARVI: with intensification of catarrhal and intoxication syndromes and atypical changes in laboratory parameters. All that complicates clinical and laboratory diagnosis and leads to incorrect administration of starting etiotropic therapy. Meglumine sodium succinate infusion crystalloid polyionic solution can be recommended for inclusion in the treatment program for patients with DF due to its complex mechanism of action (elimination of intoxication and normalization of metabolism at the cell level).

## CRediT authorship contribution statement

**D.V. Preobrazhenskaia:** Writing – original draft, Investigation, Formal analysis, Data curation, Conceptualization. **E.V. Melekhina:** Writing – review & editing, Validation, Supervision, Formal analysis, Data curation. **Zh.B. Ponezheva:** Writing – review & editing, Validation, Supervision, Formal analysis, Conceptualization.

## Declaration of competing interest

The authors declare that they have no known competing financial interests or personal relationships that could have appeared to influence the work reported in this paper.
